# Distinguishing Odors with High Temporal Precision

**DOI:** 10.1371/journal.pbio.1002022

**Published:** 2014-12-16

**Authors:** Janelle Weaver

**Affiliations:** Freelance Science Writer, Carbondale, Colorado, United States of America

**Figure 1:**
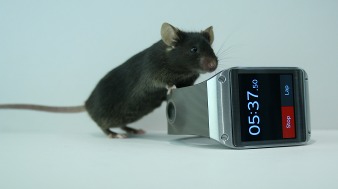
Mice can discriminate dynamics in the olfactory bulb with high temporal precision. *Photo credit: Guillermo Coronas-Sámano and Justus V. Verhagen*.

Every time you inhale the savory aromas of a home-cooked meal or the pungent smell of strong perfume, different spatial patterns of activity are evoked in odor-processing regions of the brain. Moreover, different odors elicit patterns of activity that evolve in different ways over time. For example, the timing of neuronal activity in a brain region called the olfactory bulb, relative to when an animal sniffs, conveys important information about odors. Yet relatively little is known about the behavioral relevance of the timing information generated by patterns of activity in the olfactory bulb.

In a study published this week in *PLOS Biology*, Justus V. Verhagen of the John B. Pierce Laboratory and his collaborators provide evidence that the mammalian olfactory system is capable of very high rates of transmission of transient information. Using an innovative optogenetics approach, they found that mice can precisely discriminate virtual odors associated with patterns of neuronal activity that differed by as little as 13 ms. According to the authors, the ability to rapidly and accurately recognize odors could provide a survival advantage, putting evolutionary pressure on the brain to maximize the use of odor information at the earliest stages of sensory processing.

To precisely control the timing of neuronal activity produced by virtual odors, Verhagen and his team used genetically engineered mice that expressed channelrhodopsin 2 (a light-sensitive protein originally isolated from the alga *Chlamydomonas reinhardtii* that functions as an ion channel) in a subset of olfactory bulb neurons called mitral/tufted cells. Exposure to light opened channelrhodopsin 2, allowing ions to flow inside and excite these cells. Using a novel custom-designed light projector, the researchers projected high-resolution, sniff-triggered movies onto the olfactory bulb to activate the cells and thereby stimulate the perception of virtual odors. All of the movies consisted of the same spatial pattern of eight light spots. However, in static movies, all eight spots were projected simultaneously onto the olfactory bulb, whereas in dynamic movies, one set of four spots was presented before the second set of four spots. The mice were trained to discriminate between the virtual odors by licking a spout for a water reward in response to stimulation with dynamic movies but not in response to stimulation with static movies.

The researchers set out to determine the threshold at which mice could accurately discriminate between the virtual odors based on neuronal activity in the olfactory bulb. To do so, they systematically decreased the delay between the two sets of four spots in the dynamic movie. When the two sets of spots were separated by 13 ms, the mice still achieved an accuracy of more than 75%. The findings highlight the impressive temporal precision of the olfactory system, which is comparable to the sense of touch and much higher than the sense of taste.

To establish the biological relevance of this ability to detect timing differences, the researchers created movies based on recordings of neuronal activity in the olfactory bulb of a mouse exposed to a fruity odor. These virtual odor movies were then projected onto the olfactory bulb of the genetically engineered mice. The animals were able to distinguish between these dynamic movies and static virtual odor movies that produced the same spatial pattern of neuronal activity but contained no timing information. To assess whether this temporal precision depended on the timing of neuronal activation relative to sniffing, the researchers introduced a variable jitter, ranging from zero to 50 ms, after the mice sniffed and before the movie played. The performance of the mice was unaffected by the jitter, suggesting that the temporal patterns of neuronal activation on their own can support precise odor discriminations, independent of the sniff cycle.

Taken together with past research, these new findings show that the timing of neuronal activation relative to sniffing, as well as the temporal dynamics of activity within the olfactory bulb, can both be used by mice to distinguish odors with a comparably high level of temporal precision. The study sheds new light on the ability of the olfactory system to perform very fast, information-rich computations on a vast number of odors to guide animal behavior. More broadly, the research demonstrates that the olfactory system can be used as a model system to explore how brain circuits encode complex dynamic patterns.

**Rebello MR, McTavish TS, Willhite DC, Short SM, Shepherd GM, et al. (2014) Perception of Odors Linked to Precise Timing in the Olfactory System.** doi:10.1371/journal.pbio.1002021

